# Palmitoylated mNeonGreen Protein as a Tool for Visualization and Uptake Studies of Extracellular Vesicles

**DOI:** 10.3390/membranes10120373

**Published:** 2020-11-27

**Authors:** Magda Wąchalska, Michał Rychłowski, Kinga Grabowska, Kinga Kowal, Magdalena Narajczyk, Krystyna Bieńkowska-Szewczyk, Andrea D. Lipińska

**Affiliations:** 1 Laboratory of Virus Molecular Biology, Intercollegiate Faculty of Biotechnology, University of Gdańsk, Abrahama 58, 80-307 Gdańsk, Poland; magda.wachalska@phdstud.ug.edu.pl (M.W.); michal.rychlowski@biotech.ug.edu.pl (M.R.); kinga.grabowska@biotech.ug.edu.pl (K.G.); kinga0909@vp.pl (K.K.); krystyna.bienkowska-szewczyk@biotech.ug.edu.pl (K.B.-S.); 2 Laboratory of Electron Microscopy, Faculty of Biology, University of Gdańsk, Wita Stwosza 59, 80-308 Gdańsk, Poland; magdalena.narajczyk@biol.ug.edu.pl

**Keywords:** exosomes, melanoma EVs, fluorescent labeling, mNeonGreen, palmitoylation

## Abstract

Extracellular vesicles (EVs) are membranous nanoparticles released by cells as vital mediators of intercellular communication. As such, EVs have become an attractive target for pathogens and cancer cells, which can take control over their cargo composition, as well as their trafficking, shaping the pathogenesis. Despite almost four decades of research on EVs, the number of specific and efficient EV labeling methods is limited, and there is still no universal method for the visualization of their transport in living cells. Lipophilic dyes that non-specifically intercalate into the EVs membranes may diffuse to other membranes, leading to the misinterpretation of the results. Here, we propose a palmitoylated fluorescent mNeonGreen (palmNG) protein as an alternative to chemical dyes for EVs visualization. The *Branchiostoma lanceolatum*-derived mNeonGreen is a brighter, more stable, and less sensitive to laser-induced bleaching alternative to green fluorescent protein (GFP), which makes it a more potent tag in a variety of fluorescence-based techniques. A palmNG-expressing stable human melanoma cell line was generated using retrovirus gene transfer and cell sorting. This protein partially localizes to cellular membranes, and can be detected inside size-exclusion (SEC)-purified EVs. With the use of flow cytometry and fluorescent confocal microscopy, we performed qualitative and quantitative analyses of palmNG-EVs uptake in recipient human hepatoma cells, in comparison to PKH67-labeled vesicles. Our findings confirm that membrane-embedded mNeonGreen can be successfully applied as a tool in EVs transfer and uptake studies.

## 1. Introduction

Extracellular vesicles (EVs) are small membranous nanoparticles released by cells as vital mediators of intercellular communication. They are composed of nucleic acids, proteins, metabolites, and signaling molecules encapsulated within a lipid bilayer [1]. Based on their size, density, and origin, several types of EVs can be distinguished. Exosomes are vesicles of endosomal origin that range from 30 to 150 nm in diameter. They are generated by the inward budding of an endosomal luminal membrane, leading to the formation of intraluminal vesicles (ILVs) within multivesicular bodies (MVBs). The subsequent fusion of MVBs with the plasma membrane results in the extracellular release of ILVs as exosomes. Microvesicles, also known as ectosomes, are larger vesicles, 100 nm–1 µm in diameter, and their biogenesis involves direct budding from the plasma membrane. Apoptotic bodies, released during apoptosis, represent the largest type of EVs, ranging from 50 nm up to 5 µm in diameter [2]. EVs are secreted to various biological fluids—including blood, milk, saliva, and urine—as well as to cell culture supernatants in vitro [3,4]. Initially, they were considered to be a cellular waste, but currently, EVs have been acknowledged for their significant role in physiological processes such as stem cell differentiation, acquired and adaptive immune response or autophagy [5,6,7], and pathologies like tumor invasion and metastasis, neurodegenerative diseases, and pathogen infections [8,9,10,11].

The transport and internalization/uptake of EVs by the recipient cells as a part of intercellular communication have been extensively studied in recent years. Despite almost four decades of research on EVs, the number of specific and efficient EV-labeling methods is limited, and there is still no universal approach for the visualization of their transport in living cells or in vivo. Fluorescent tagging has been the most common method to follow the vesicles. Lipophilic dyes—like the PKH family (with PKH26-red fluorescence, and PKH67-green fluorescence), Dil, or DiD—consist of a highly fluorescent polar head attached to a long aliphatic tail, which intercalates into all kinds of lipid bilayers. However, it has been reported that those dyes may alter the size of vesicles [12], form large aggregates or nanoparticles with similar size to EVs [13], or diffuse to other cellular membranes [14] leading to the misinterpretation of the results. The approach utilizing the conjugation of tetraspanins enriched in extracellular vesicles—for instance, CD63 [15] or CD9 [16]—with fluorescent reporters such as enhanced green fluorescent protein (eGFP) limits the analysis to the subpopulation of vesicles enriched with the tagged marker. As shown in previous studies, the uptake and subsequent trafficking of EVs in a recipient cell depend on the shape, size, surface molecule composition, and hydrophobicity of EVs [17]; therefore, an approach that leaves those properties of the extracellular vesicles unaffected is desirable.

S-palmitoylation is a reversible attachment of palmitic acid to the cysteine residues of a substrate protein via a thioester bond, which—as a result—can anchor the target protein in a cell membrane [18]. A family of conjugating enzymes, palmitoyl acyltransferases (PATs), which contain a conserved aspartate–histidine–histidine–cysteine (DHHC) motif, have been identified quite recently in the human genome, and currently at least 23 members are known [19]. Since this post-translational modification allows for the association of proteins with cellular membranes, a palmitoylated fluorescent reporter has been suggested to be a useful tool in uptake studies of EVs [20].

Here, we propose a palmitoylated fluorescent mNeonGreen (palmNG) protein as an alternative for chemical dyes for the visualization of EVs trafficking. mNeonGreen is a monomeric variant of a yellow-green fluorescent protein derived from a marine lancelet, *Branchiostoma lanceolatum* [21], characterized as being brighter, more stable, and less sensitive to laser-induced bleaching than GFP, which makes it a more potent tag in a variety of fluorescence-based techniques.

## 2. Materials and Methods

### 2.1. Cells

Human melanoma Mel JuSo cells (MJS, a kind gift from Dr. Emmanuel Wiertz, University Medical Center Utrecht, Utrecht, The Netherlands) were cultured in RPMI 1640 (Corning, Corning city, NY, USA) supplemented with 10% fetal bovine serum (FBS, Thermo Scientific, Waltham, MA, USA) and Antibiotic Antimycotic Solution (Thermo Scientific). GP2-293 cells (Takara/Clontech, Kusatsu, Japan), used for retrovirus production, were cultured in Iscove’s modified Dulbecco’s medium (IMDM, Lonza, Basel, Switzerland), supplemented as above. Human hepatocellular carcinoma Huh-7 cells (a kind gift from Dr. Arvind Patel, University of Glasgow, Glasgow, UK) were cultured in Dulbecco’s modified Eagle’s medium (DMEM, Corning), supplemented as above.

### 2.2. Generation of a Stable Cell Line Expressing Palmitoylated mNeonGreen

A synthetic gene coding for mNeonGreen (based on GeneBank accession number: KC295282.1, codon-optimized) was cloned into the pJET1.2 plasmid (Thermo Scientific). The S-palmitoylation signal MLCCMRRTKQ was introduced at the N-terminus of mNeonGreen by sequential PCR with the proofreading WALK (Pwo) polymerase (A&A Biotechnology, Gdynia, Poland) and the following primers: forward F1: 5’-GAGACGCACAAAGCAGGTGAGCAAGGGC-3’; F2: 5’-GGATCCACC**ATG**CTATGTTGCATGAGACGCAC-3’; and reverse: 5’-GAATTCTTACTTGTACAGCTCGTCCATGC-3’ (the start codon in bold). The BglII-digested and Klenow fragment-modified sequence coding for palmitoylated mNeonGreen (palmNG) was cloned into the HpaI-digested pLNCX retroviral vector (Takara/Clontech).

The retroviral packaging system was used to obtain the recombinant retroviruses. GP2-293 packaging cells (Takara/Clontech) were cotransfected with the transfer plasmids pLNCXpalmNG and pCMV-VSV-G (Cell Biolabs, San Diego, CA, USA) for pseudotyping, using a CalPhos mammalian transfection kit (Takara/Clontech). Twenty-four hours after the transfection, the medium was refreshed. Virus-containing supernatants were collected after 48h, concentrated with PEGit (System Biosciences, Palo Alto, CA, USA), and used for the transduction of MJS cells in the presence of 0.01 mgmL^−1^ polybrene (Merck/Sigma-Aldrich, Darmstadt, Germany). MJS palmNG-positive cells were sorted using a FACS Calibur flow cytometer with the sorting option (Becton Dickinson, Franklin Lakes, NJ, USA).

### 2.3. Antibodies

The antibodies used for the immunoblotting were: mouse anti-CD63 (clone MX-49.129.5, Santa Cruz Biotechnology, Dallas, TX, USA), mouse anti-CD9 (clone MCA-469GA, Bio-Rad/AbD Serotec, Hercules, CA, USA), mouse anti-Alix (clone 3A9, Santa Cruz Biotechnology), mouse anti-HLA-DR (clone L243, Santa Cruz Biotechnology), rabbit anti-flotillin-2 (C42A3, Cell Signaling Technology, Danvers, MA, USA), goat anti-calnexin (C-20, Santa Cruz Biotechnology), rabbit anti-Tom40 (H-300, Santa Cruz Biotechnology), and mouse anti-NeonGreen (32F6, Chromotek, Planegg, Germany). Goat anti-mouse horseradish peroxidase (HRP)-conjugated IgG, donkey anti-rabbit HRP-conjugated IgG, and donkey anti-goat HRP-conjugated IgG (Jackson Immunoresearch, West Grove, PA, USA) were used as secondary antibodies in the immunoblotting. The probes used for immunofluorescence were: rabbit anti-β-catenin antibody (H-102, Santa Cruz Biotechnology), goat anti-rabbit AlexaFluor 546-conjugated IgG (Thermo Scientific), MitoTracker Red (Thermo Scientific), and Hoechst 33,342 (Thermo Scientific).

### 2.4. Isolation of Extracellular Vesicles

The EVs were isolated by ultrafiltration with size-exclusion chromatography (SEC) according to the protocol described in [22] and [23], with minor modifications [24]. MJS cells were plated on four T-175 culture flasks. When the confluence of the cells was 70%, the medium was replaced with the serum-free medium Hybridomed DIF-1000 (Biochrom, Berlin, Germany) supplemented with Antibiotic Antimycotic Solution, in order to avoid contamination with serum-derived products. After 48 h, the media for the EVs isolation were collected and precleared by centrifugation for 10 min at 300× *g*, 20 min at 500× *g*, and 20 min at 2000× *g*, followed by the filtration of the supernatants using 0.45 µm filters (polyvinylidene fluoride membrane) in order to remove the larger vesicles. The samples were concentrated by ultrafiltration using Amicon^®^ Ultra-15 Centrifugal Units 100-K (Merck, Darmstadt, Germany) in order to reduce the volume to 1 mL, and were subsequently used for the SEC isolation of the EVs. The SEC column (10 mL plastic syringe, Becton Dickinson) was stacked with Sepharose CL-4B (GE Healthcare, Chicago, IL, USA) suspension in 0.02 µm-filtered PBS (the column bed dimentions were 16 mm × 62 mm). The supernatant was loaded onto the column, followed by separation with the filtered PBS, and fractions of 0.5 mL were collected.

### 2.5. Immunoblotting

Cell lysates were obtained in the Cell Lytic M buffer (Merck/Sigma-Aldrich, Darmstadt, Germany). EVs were lysed in the RIPA lysis buffer (150 mM NaCl, 1% Nonidet P-40, 50 mM Tris-HCl, pH 7.6, 0.1% SDS, 5 mM EDTA, 0.5% sodium deoxycholate). The complete mini protease inhibitor cocktail (Roche, Basel, Switzerland) was added to both buffers to prevent proteolysis. The total protein concentration was quantified using the BCA Protein Assay QPRO–BCA (Cyanagen, Bologna, Italy). The cell and EVs lysates were separated in SDS-PAGE and immunoblotted as described previously [25]. CD63 was analyzed in non-reducing conditions, according to [26]. For the dot blot, EVs were spotted on a nitrocellulose membrane in a dose range. The membranes were blocked with 5% skim milk in TBS (10 mM Tris-HCl, pH 8.0, 150 mM NaCl) or TBST (TBS, 0.1% Tween 20) for the conditions in the presence of a detergent. Subsequently, the membranes were incubated with anti-NeonGreen or anti-CD9 primary antibodies in 2.5% milk-TBS or milk-TBST, followed by incubation with HRP-conjugated secondary antibodies in 2.5% milk-TBS or milk-TBST. The signal was detected with the Super Signal West Pico Plus Chemiluminescent Substrate (Thermo Scientific).

### 2.6. Immunofluorescence

MJSpalmNeonGreen cells were grown on microcover glass, fixed with 4% paraformaldehyde in PBS, and permeabilized with 0.2% Triton X-100 in PBS. Next, they were washed in PBS, incubated with the rabbit anti-β-catenin antibody, followed by goat anti-rabbit AlexaFluor 546-conjugated IgG, and analyzed using a Leica TCS SP8X confocal laser scanning microscope (Leica Microsystems, Wetzlar, Germany). The degree of the co-localization was quantified using the overlap coefficient with Leica Application Suite X software. For the analysis of the EVs in solution, 5 µL droplets of the EVs preparations were placed on a microscope slide and analyzed with Leica TCS SP8X.

### 2.7. Analysis of EVs Uptake by Recipient Cells

For the flow cytometry, Huh-7 cells were plated on a 12-well plate. When the confluence of the cells was 80%, 35–70 μg of EVs, or PBS as a control, were added to cells in a final volume of 600 μL of Hybridomed DIF-1000 medium and incubated for 3 h or 6 h at 37 °C. After the indicated time, the cells were washed 3 times with PBS, trypsinized, collected into 250 μL PBS, and analyzed by flow cytometry using Guava easyCyte 6, software version GuavaSoft 3.1.1 (Merck, Darmstadt, Germany).

For the fluorescence confocal microscopy, Huh-7 cells were plated on a µ-Slide 8-well chamber slide (ibidi, Gräfelfing, Germany). When the confluence of the cells was 60%, 15 μg of EVs were added to the cells in a final volume of 200 μL Hybridomed DIF-1000 medium, and incubated for 6 h at 37 °C. After the indicated time, the cells were washed 3 times with PBS and fresh Hybridomed DIF-1000 media with MitoTracker Red and Hoehst 33342. Micrographs and confocal Z-stack images for the 3D reconstruction were captured with the Leica TCS SP8X confocal laser scanning microscope under environmental control at 37 °C and 5% CO_2_.

### 2.8. Transmission Electron Microscopy (TEM)

TEM was performed as described previously [24]. Briefly, EVs samples were adsorbed on formvar–carbon-coated 200-mesh nickel grids. Negative staining was performed with 2% uranyl acetate. The grids were analyzed using a transmission electron microscope, Tecnai G2 Spirit BioTWIN (FEI Inc., Hillsboro, OR, USA), at 120 kV, at the Laboratory of Electron Microscopy, University of Gdańsk.

### 2.9. PKH67 Labeling of the EVs

MJS-derived EVs were labeled with PKH67 (Merck/Sigma-Aldrich, Darmstadt, Germany) according to the manufacturer’s protocol. The EVs sample was diluted 1:10 in diluent C, followed by the addition of PKH67 dye (1:1000), and incubated for 5 min at room temperature in the dark. The reaction was stopped by adding 1% bovine serum albumin in PBS. In order to clear the EVs from the redundant dye, the stained EVs sample was diluted with PBS, and subsequently ultrafiltrated using Amicon^®^ Ultra-15 Centrifugal Units 100-K.

### 2.10. Nanoparticle Tracking Analysis (NTA)

In order to determine the concentration and the size distribution of the EVs, nanoparticle tracking analysis (NTA) was performed using Nanosight NS300 (Malvern Instruments, Salisbury, UK). The EVs were diluted in 0.02 µm-filtered PBS to a concentration between 10^8^ and 10^9^ particles mL^−1^. Using NTA V3.3 software, three 60s videos were recorded and analyzed per sample, with the camera at level 16, and a detection threshold of 5.

## 3. Results

### 3.1. Palmitoylated mNeonGreen Partially Localizes to Cellular Membranes

**Figure 1:**
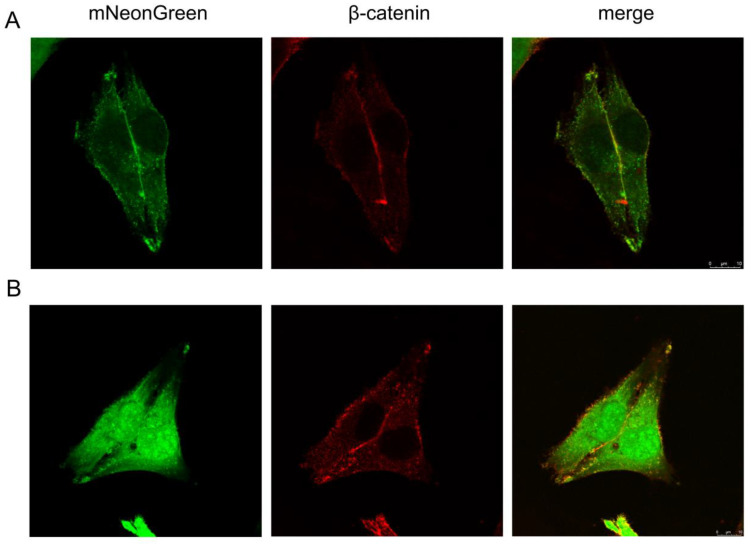
Palmitoylated mNeonGreen (palmNG) partially localizes to cellular membranes. MJSpalmNG cells were fixed, permeabilized, and stained with anti-β-catenin antibody, followed by AlexaFluor 546-conjugated IgG. The colocalization of palmNG and β-catenin was analyzed using fluorescent confocal microscopy. (**A**,**B**) show representative images of two different palmNG localization patterns in MJSpalmNG cells.

In order to obtain a cell line producing fluorescent EVs, a sequence coding for the MLCCMRRTKQ S-palmitoylation signal—designed based on the sequence of neuronal growth cone-associated protein (GAP43)—was cloned upstream and in frame of the mNeonGreen gene. mNeonGreen was chosen as a reporter due to its advantages over eGFP. The GAP43 palmitoylation signal has been previously applied by others to drive the membrane localization of chloramphenicol acetyltransferase [27], or fluorescent dTomato and GFP [20]; palmGFP has been used to label EVs [20]. With the use of a retroviral gene transfer and cell sorting, we obtained a stable human melanoma cell line (MJS) with the constitutive palmNG expression. In order to determine the cellular localization of palmitoylated mNeonGreen (palmNG), the cells were stained with anti-β-catenin antibody, and the colocalization of palmNG with β-catenin—a cell membrane-resident protein—was analyzed by confocal laser scanning microscopy. Surprisingly, we could observe two different localization patterns of the fluorescent reporter. In the first pattern, palmNG localized, predominantly, to the cell membranes and various intracellular vesicles (Figure 1A). However, we could also notice cells with palmNG distributed in the entire cell, resembling the endoplasmic reticulum (ER) staining, and also including the nucleus, in addition to the plasma membrane (Figure 1B). In both cases, the overlap coefficient indicated significant co-localization of palmNG and β-catenin.

### 3.2. Palmitoylated mNeonGreen Localizes to the EVs

**Figure 2:**
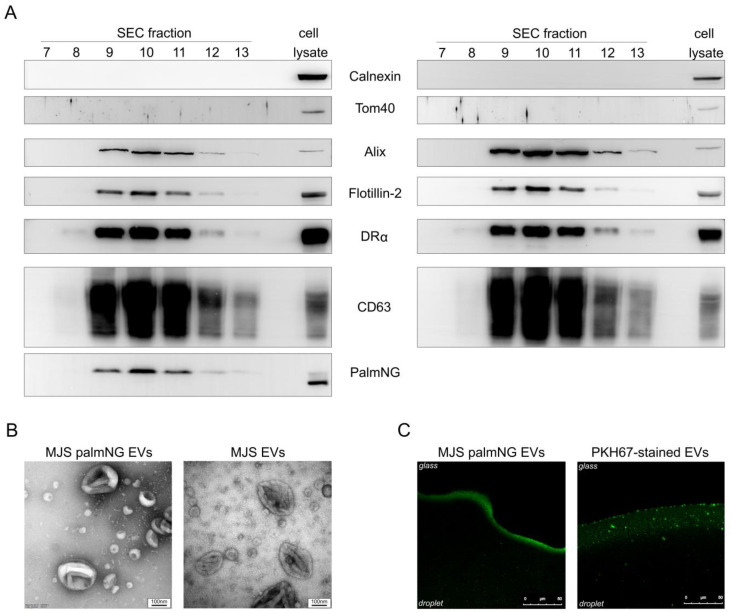
Characterization of size exclusion chromatography (SEC)-purified EVs. EVs from MJSpalmNG (left panels) and MJS (right panels) were isolated by SEC. (**A**) Selected fractions (20 µL) were analyzed by SDS-PAGE and immunoblotting for the presence of EVs markers with the use of anti-Alix, anti-flotillin-2, anti-DRα, anti-CD63 antibodies, and for the presence of cellular markers with the use of anti-calnexin and anti-Tom40 antibodies. The presence of palmNG in the EVs-enriched fractions was confirmed with anti-NeonGreen antibodies. A cell lysate from the EVs donor cell line was used as a control. (**B**) Representative transmission electron microscopy images of EVs from pooled EVs-containing fractions (F9-F11); scale bar 100 nm. (**C**) Fluorescent EVs in solution (a droplet on a coverglass). The images of the MJSpalmNG EVs or PKH67-stained MJS EVs were captured using a confocal laser scanning microscope.

EVs secreted by MJS and MJSpalmNG were isolated and purified by ultrafiltration with size exclusion chromatography (SEC). Selected fractions from the SEC were analyzed by SDS-PAGE and immunoblotting (Figure 2A). EVs markers Alix (90 kDa), flotillin-2 (50 kDa), HLA-DRα (MHC class II) (35 kDa), and CD63 (26-60 kDa), detected by specific antibodies, were present predominantly in fractions 9–11. The presence of mNeonGreen (27 kDa) in the EVs-enriched fractions was confirmed with specific anti-NeonGreen antibodies. Cellular markers calnexin (90 kDa), which is an ER-resident protein, and Tom40 (40 kDa)—a mitochondria outer membrane-associated protein—were detected only in a cell lysate, not in the EVs fractions, which confirms the purity of the isolated vesicles. Interestingly, we could detect mNeonGreen in cell lysates as a double band (25 and 27 kDa), and only the higher molecular-weight form seemed to be present in the EVs. EVs-enriched fractions (9–11) were pooled and used for further analysis. The morphology of the EVs analyzed by transmission electron microscopy (TEM) revealed their typical cup-shaped morphology (Figure 2B), whereas nanoparticle tracking analysis (NTA) determined the size of the EVs ranging from 40 to 240 nm, with an average diameter of 118 nm, which was in line with the TEM observations.

PKH67 is one of the most commonly-used lipophilic dyes to study EVs transfer between cells; however, it was reported to increase the size of the labeled EVs and form nanoparticles or micelles, as well as aggregates [12,13]. In order to examine whether palmNG labeling might similarly affect EVs, we compared the homogeneity of the fluorescent signal from vesicles in solution by confocal laser scanning microscope and NTA. As depicted in Figure 2C, palmNG EVs exhibited a visually-homogenous population of vesicles based on the analysis of the green fluorescence signal, while the PKH67-stained sample could be characterized with the presence of smaller and larger structures, possibly aggregates. These observations were supported by the NTA, which indicated the presence of an additional population of larger structures—ranging from 250 to 450 nm—and a minor fraction of 640 to 700 nm in diameter for the PKH-stained vesicles, which was in agreement with [12]. Nevertheless, the mean diameter of the PKH67 EVs samples (106.5 +/−2.6 nm), as revealed by the NTA, was similar to that of the palmNG-labeled EVs (118.6 +/−1.4 nm), and the mode value was equal to 78 nm for both samples.

### 3.3. Palmitoylated mNeonGreen Is Present inside EVs

**Figure 3:**
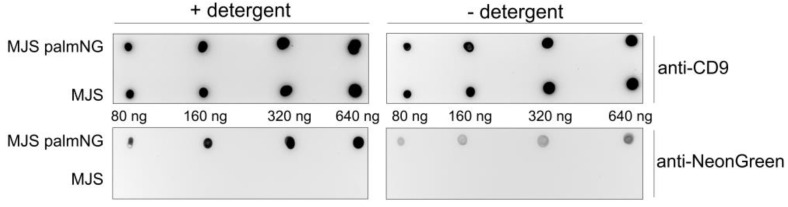
Localization of palmNG in EVs. Various amounts of EVs from MJS or MJSpalmNG cells were dot-blotted in a dose range on a nitrocellulose membrane and analyzed by immunoblotting with the use of anti-mNeonGreen or anti-CD9 antibody in the presence or absence of a detergent.

The orientation of the palmNG in the EVs membrane may be important for the subsequent studies; therefore, we tested various doses of the isolated EVs samples (from MJS or MJSpalmNG) by dot-blot in the presence or absence of a detergent (Tween 20). The detergent could enhance the immunoblotting detection of mNeonGreen (Figure 3, lower panels). This observation suggests the localization of the fluorescent reporter in the inner space of the EVs, which required detergent treatment to allow the access of antibodies to the internally-localized palmNG. As a control, we detected the CD tetraspanin CD9, which has the epitope recognized by the specific antibodies localized outside of EVs. In this case, the signal from the immunoblotting detection seemed to be equally strong, regardless of the detergent (Figure 3, upper panels).

### 3.4. PalmNG EVs Internalized by Huh-7 Cells Localize Predominantly to the Perinuclear Space

**Figure 4:**
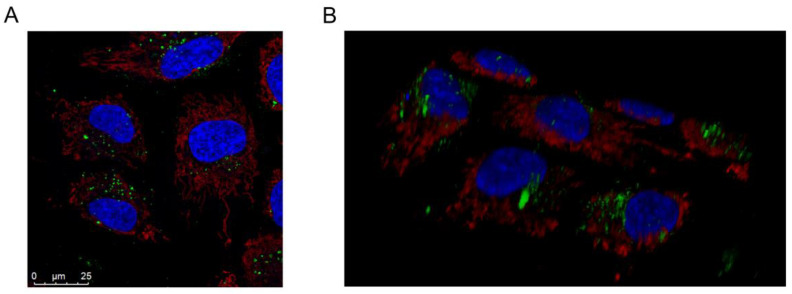
PalmNG EVs internalized by Huh-7 cells localize predominantly to the perinuclear area. Huh-7 cells were incubated with MJSpalmNG-derived EVs for 6 h at 37 °C, followed by mitochondria and nucleus staining with MitoTracker Red and Hoehst 33342, respectively, and imaging by confocal microscopy. (**A**) A representative single Z-stack image; (**B**) a 3D reconstruction of the confocal Z-stack images. See Supplementary Video S1 for the reconstructed confocal Z-stack image animation.

Next, we investigated the application potential of palmNG-labeled vesicles in EVs transfer and uptake studies. Such studies should be performed with care to exclude the fluorescence signal representing the vesicles only attached to the cell surface and not internalized by the recipient cells. Therefore, we analyzed palmNG-EVs uptake by confocal laser scanning microscopy. Since one of the most common targets of melanoma cancer cells for metastasis is the liver [28], we selected the human hepatocellular carcinoma cell line (Huh-7) as EVs recipients. Huh-7 cells were incubated with palmNG EVs in an EVs-depleted medium for 6 h under environmental conditions. After thorough washing with PBS, we stained the cell mitochondria and nuclear DNA in order to visualize those cellular compartments. Z-stack images demonstrated the perinuclear localization of the internalized palmNG EVs (a representative image is given in Figure 4A), while the 3D reconstruction of the confocal images confirmed the intracellular localization of the fluorescent signals in the vesicular structures concentrating rather in the perinuclear space than in the plasma membrane-proximal area (Figure 4B, and the 3D animation is given in Supplementary Video S1).

### 3.5. The Uptake of PalmNG-EVs by Huh-7 Cells Is Time- and Dose-Dependent

**Figure 5:**
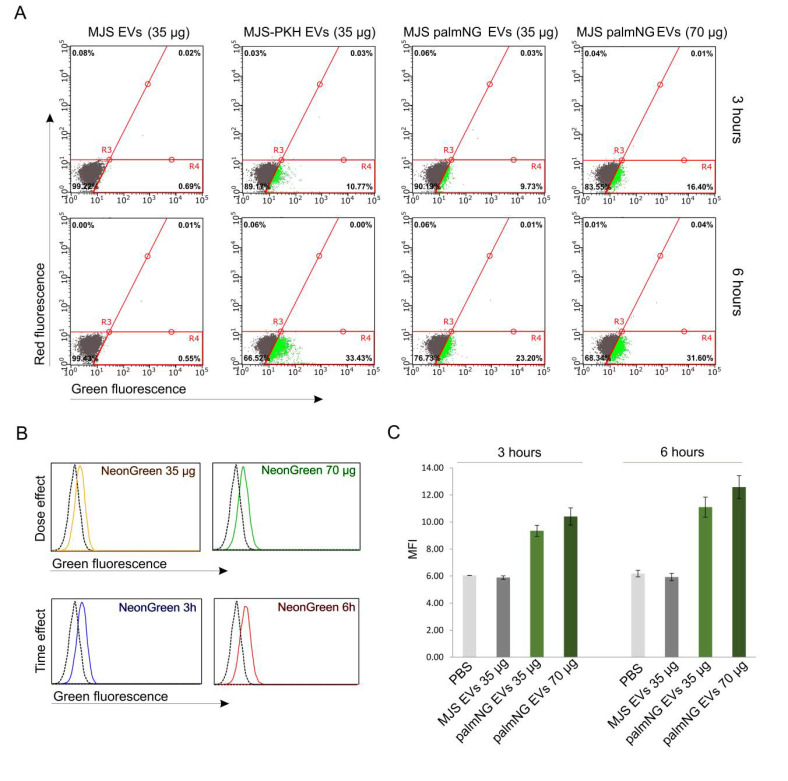
Time- and dose-dependent palmNG-EVs uptake by Huh-7 cells. Huh-7 cells were incubated with MJSpalmNG-derived EVs in a dose range. MJS-derived EVs or MJS-derived EVs stained with PKH67 were used as controls. After the indicated time points, the cells were collected and analyzed using flow cytometry in order to assess the EVs uptake. (**A**) Green fluorescence-positive cells were gated as the green cell population. (**B**) Representative histograms show a dose-dependent and time-dependent shift in the GFP fluorescence of the internalizing cells. The black lines represent MJS-derived EVs; the colored lines represent palmNG EVs. (**C**) The mean fluorescence intensities (MFI) of the internalized EVs from a duplicate analysis are represented as bars with standard deviations.

Gaining evidence that the green fluorescence signal comes only from the internalized vesicles, we performed a quantitative analysis of the EVs uptake. Huh-7 cells were incubated with different amounts of palmNG EVs in EVs-depleted media for 3 or 6 h under environmental conditions. PBS, MJS-derived EVs, or PKH67-stained EVs were used as controls. After the indicated time, the cells were collected and the vesicle internalization was assessed using flow cytometry. The cells incubated with PBS and MJS-derived EVs were gated as a negative population, whereas the cells exhibiting green fluorescence were depicted as a green population (Figure 5A). We observed this internalizing population of cells growing with time and the dose of palmNG-EVs (9.7% or 16.4% in a dose range of 35 μg and 70 μg, respectively after 3 h and 23.2% or 31.6% after 6 h). It is worth noticing that there was a high degree of homogeneity of the green fluorescence intensity. We could observe a uniform shift in green fluorescence (Figure 5A,B) indicating that all of the cells internalized similar amounts of EVs. A slightly different observation could be made for the PKH67-stained EVs-internalization, depicting a cell population with a more heterogeneous green fluorescence signal (Figure 5A). The time- and dose-dependent manner of the fluorescent EVs uptake was also presented as an increase of the mean fluorescence intensity (MFI) of the whole cell population (Figure 5C).

## 4. Discussion

In this communication, we proposed a palmitoylated mNeonGreen fluorescent protein as a tool for the visualization of EVs transfer between cells, and we compares it to the PKH67 dye commonly used for this purpose. We designed this system in order to facilitate our research on the uptake of EVs incorporating herpesvirus-encoded microRNA.

We have previously used PKH67-labeled vesicles; however, we could observe relatively large fluorescent structures in the experiments, even when the samples were filtered using 0.45 μM membranes. Such structures were also demonstrated in this study, especially in the fluorescence analysis of the samples in droplets (Figure 2C). Interestingly, our NTA analysis of PKH67-labeled EVs revealed a similar mean diameter of the vesicles. Nevertheless, small populations of large EVs ranging from 250 to 450 nm, and even up to 700 nm were noted. In this respect, palmNG-labeled EVs were more uniform in the analyses. The potential of PKH dyes to increase the size of the EVs and dye clusters or aggregate formations has been reported [12,13,29]. Several studies have associated those properties with the altered distributions [30] and shortened circulation half-lives of such EVs in vivo, probably because of the activation of the complement system [31]. What is more, the strong non-covalent interaction of the PKH aliphatic tail with the lipid bilayer may promote the long-lasting retention of a dye in the membrane. PKH dyes were shown to exhibit an in vivo half-life ranging from 5 to 100 days [32], which extremely exceeds the half-life of EVs, which are estimated to reach up to 24 h in in vitro cultures [20], or from 30 min to 6 h in vivo when biotinylated EVs were administered intravenously to various tissues [14]. Hence, these lipophilic dyes would not reflect the real EVs half-life circulation, but rather prevail in cellular membranes and possibly diffuse to other cellular compartments.

Those concerns persuaded us to switch to a fluorescent protein reporter. We chose mNeonGreen as a three–five times brighter, more stable, and less sensitive to laser-induced bleaching alternative for eGFP (see the Supplementary Figure S2 for comparison). Its excitation and emission spectra slightly differ from eGFP, though mNeonGreen is compatible with GFP-dedicated filters, and was successfully used in vitro as well as in vivo [21,33]. The GAP43 S-palmitoylation signal represents one of the best-documented sequences for the anchoring of fluorescent reporters (eGFP and dTomato) in the EVs membrane [20,27]. The palmitoylation of both cysteines in this sequence at the endoplasmic reticulum-Golgi intermediate compartment drives GAP43 protein to the inner surface of the plasma membrane of growing axons (GAP43 is reviewed in [34]). Another option for the anchoring of a reporter in the EVs membrane could be the use of a heterologous transmembrane domain, as in the *Gaussia* luciferase construct reported by [35].

We chose human melanoma cells as EVs donors, since they have been reported to secrete more EVs than healthy melanocytes, and because melanoma-derived EVs were shown to take part in metastasis by educating bone marrow cells [10,36]. The stable integration of the palmNeonGreen gene in the genome of the producer cells resulted in a typical membranous localization of the green fluorescence signal; however, we could also observe cells with mNeonGreen distributed in the entire cell, including the nucleus and, possibly, the ER (Figure 1). We confirmed this dual pattern also in HEK293T and Huh-7 cells transfected with the palmNG reporter. It differs from the localization reported for palmGFP or palmdTomato [20]. It seems that the diverse subcellular localization corresponded with the detection of two mNeonGreen forms in the immunoblotting (Figure 1A), and indicated efficient palmitoylation in only a fraction of the protein. One possible explanation could be a diverse genomic integration of a retrovirus that, in some cells, resulted in high expression from the human cytomegalovirus promoter. The subsequent production of large amounts of the fluorescent reporter could overload the cellular palmitoylation machinery. PATs were shown to localize in several membranous compartments [37], but their relevant enzymatic activity was suggested to prevail in Golgi [38]. Therefore, it is reasonable to speculate that the excess of palmNG is distributed elsewhere in the cell. What is more, palmitoylation is the only known reversible lipid modification and, in contrast to the spatial organization of palmitoylation, depalmitoylation may take place in the entire cell. Such seconds-long reactions have been reported for GAP43 [39]. PATs seem to lack specific consensus sequence specificity, and only the cysteine residue is crucial for the thioester bond formation [40,41]. Although the flanking sequence does not have an impact on the substrate recognition, it may be responsible for the accessibility to the thiol group of the cysteine residue, which is dictated by the substrate conformation. GFP-like fluorescent proteins, even evolutionarily-distinct ones, like mNeonGreen and GFP, have a conserved, characteristic, β-barrel structure [21,42]. The N-termini of the two proteins differ slightly (Supplementary Figure S2), which may account for the accessibility of the two reporters to PATs. Finally, some reports suggest that the insertion of short linkers between the palmitoylation signal (like the one derived from the endothelial nitric oxide synthase) and GFP can ensure proper access to PATs and membrane localization [43]. The construction of such a palmNG version should, in the future, reveal whether that approach can lead to more efficient labeling of EVs.

Importantly, despite the diverse palmNG distribution in cells, we found it incorporated in the melanoma-secreted EVs. Only the higher molecular-weight mNeonGreen form, most probably representing palmNG, could be found in EVs by immunoblotting, confirming that membrane-association is required for the incorporation of the reporter to the vesicles. The inner membrane localization of palmNG minimizes alterations to the surface composition of EVs, and helps to preserve interactions with the surface molecules of recipient cells, enabling EVs uptake or simply ligand-receptor binding. What is more, palmNG-luminal tagging does not affect the size of the EVs, as was confirmed by TEM (Figure 2B) and NTA.

By the fluorescent intraluminal tagging of EVs with palmNG, we obtained a reliable tool that allowed us to follow their uptake by recipient cells. We have shown that the internalization of melanoma-derived EVs by Huh-7 cells is a time- and dose-dependent process, during which the vesicles localized predominantly to the perinuclear area, as was consistent with other studies [44,45,46,47]. Based on the analysis of the green fluorescence signal size and distribution, we might speculate that palmNG EVs were trapped in vesicles such as endosomes, transported actively to the perinuclear region, and accumulated in larger organelles. However, the exact mechanism needs to be further elucidated, and palmNG-tagged EVs seems to be a suitable tool in these studies.

## 5. Conclusions

Even though the distribution of palmitoylated mNeonGreen and eGFP may vary in the EVs-producing cells, our findings confirm that palmNG can be successfully applied as a tool for the labeling of EVs in the transfer and uptake studies of the vesicles.

## Supplementary Materials

The following are available online at https://www.mdpi.com/2077-0375/10/12/373/s1: Video S1: reconstituted confocal Z-stack images of palmNG EVs uptake by Huh-7cells as a 3D animation; Supplementary Figure S2: comparison of mNeonGreen and eGFP reporters.

## References

[1] Théry C., Zitvogel L., Amigorena S. (2002). Exosomes: Composition, biogenesis and function. Nat. Rev. Immunol..

[2] Kalra H., Drummen G., Mathivanan S. (2016). Focus on Extracellular Vesicles: Introducing the Next Small Big Thing. Int. J. Mol. Sci..

[3] Théry C., Amigorena S., Raposo G., Clayton A. (2006). Isolation and Characterization of Exosomes from Cell Culture Supernatants and Biological Fluids. Curr. Protoc. Cell Biol..

[4] Keller S., Ridinger J., Rupp A.-K., Janssen J.W., Altevogt P. (2011). Body fluid derived exosomes as a novel template for clinical diagnostics. J. Transl. Med..

[5] Nair R., Santos L., Awasthi S., von Erlach T., Chow L.W., Bertazzo S., Stevens M.M. (2014). Extracellular Vesicles Derived from Preosteoblasts Influence Embryonic Stem Cell Differentiation. Stem Cells Dev..

[6] Robbins P.D., Morelli A.E. (2014). Regulation of immune responses by extracellular vesicles. Nat. Rev. Immunol..

[7] Baixauli F., López-Otín C., Mittelbrunn M. (2014). Exosomes and Autophagy: Coordinated Mechanisms for the Maintenance of Cellular Fitness. Front. Immunol..

[8] Caobi A., Nair M., Raymond A.D. (2020). Extracellular Vesicles in the Pathogenesis of Viral Infections in Humans. Viruses.

[9] Jurj A., Pop-Bica C., Slaby O., Ştefan C.D., Cho W.C., Korban S.S., Berindan-Neagoe I. (2020). Tiny Actors in the Big Cellular World: Extracellular Vesicles Playing Critical Roles in Cancer. Int. J. Mol. Sci..

[10] Peinado H., Alečković M., Lavotshkin S., Matei I., Costa-Silva B., Moreno-Bueno G., Hergueta-Redondo M., Williams C., García-Santos G., Ghajar C.M. (2012). Melanoma exosomes educate bone marrow progenitor cells toward a pro-metastatic phenotype through MET. Nat. Med..

[11] Hill A.F. (2019). Extracellular Vesicles and Neurodegenerative Diseases. J. Neurosci..

[12] Dehghani M., Gulvin S.M., Flax J., Gaborski T.R. (2020). Systematic Evaluation of PKH Labelling on Extracellular Vesicle Size by Nanoparticle Tracking Analysis. Sci. Rep..

[13] Pužar Dominkuš P., Stenovec M., Sitar S., Lasič E., Zorec R., Plemenitaš A., Žagar E., Kreft M., Lenassi M. (2018). PKH26 labeling of extracellular vesicles: Characterization and cellular internalization of contaminating PKH26 nanoparticles. Biochim. Biophys. Acta (BBA) Biomembr..

[14] Lai C.P., Mardini O., Ericsson M., Prabhakar S., Maguire C.A., Chen J.W., Tannous B.A., Breakefield X.O. (2014). Dynamic Biodistribution of Extracellular Vesicles in Vivo Using a Multimodal Imaging Reporter. ACS Nano.

[15] Mittelbrunn M., Gutiérrez-Vázquez C., Villarroya-Beltri C., González S., Sánchez-Cabo F., González M.Á., Bernad A., Sánchez-Madrid F. (2011). Unidirectional transfer of microRNA-loaded exosomes from T cells to antigen-presenting cells. Nat. Commun..

[16] Fabbri M., Paone A., Calore F., Galli R., Gaudio E., Santhanam R., Lovat F., Fadda P., Mao C., Nuovo G.J. (2012). MicroRNAs bind to Toll-like receptors to induce prometastatic inflammatory response. Proc. Natl. Acad. Sci. USA.

[17] Salatin S., Maleki Dizaj S., Yari Khosroushahi A. (2015). Effect of the surface modification, size, and shape on cellular uptake of nanoparticles: Cellular uptake of nanoparticles. Cell Biol. Int..

[18] Aicart-Ramos C., Valero R.A., Rodriguez-Crespo I. (2011). Protein palmitoylation and subcellular trafficking. Biochim. Biophys. Acta.

[19] Mitchell D.A., Vasudevan A., Linder M.E., Deschenes R.J. (2006). Thematic review series: Lipid Posttranslational Modifications; Protein palmitoylation by a family of DHHC protein *S*-acyltransferases: Fig. 1. J. Lipid Res..

[20] Lai C.P., Kim E.Y., Badr C.E., Weissleder R., Mempel T.R., Tannous B.A., Breakefield X.O. (2015). Visualization and tracking of tumour extracellular vesicle delivery and RNA translation using multiplexed reporters. Nat. Commun..

[21] Shaner N.C., Lambert G.G., Chammas A., Ni Y., Cranfill P.J., Baird M.A., Sell B.R., Allen J.R., Day R.N., Israelsson M. (2013). A bright monomeric green fluorescent protein derived from Branchiostoma lanceolatum. Nat. Methods.

[22] Böing A.N., van der Pol E., Grootemaat A.E., Coumans F.A.W., Sturk A., Nieuwland R. (2014). Single-step isolation of extracellular vesicles by size-exclusion chromatography. J. Extracell. Vesicles.

[23] Nordin J.Z., Lee Y., Vader P., Mäger I., Johansson H.J., Heusermann W., Wiklander O.P.B., Hällbrink M., Seow Y., Bultema J.J. (2015). Ultrafiltration with size-exclusion liquid chromatography for high yield isolation of extracellular vesicles preserving intact biophysical and functional properties. Nanomed. Nanotechnol. Biol. Med..

[24] Grabowska K., Wąchalska M., Graul M., Rychłowski M., Bieńkowska-Szewczyk K., Lipińska A.D. (2020). Alphaherpesvirus gB Homologs Are Targeted to Extracellular Vesicles, but They Differentially Affect MHC Class II Molecules. Viruses.

[25] Lipinska A.D., Koppers-Lalic D., Rychlowski M., Admiraal P., Rijsewijk F.A.M., Bienkowska-Szewczyk K., Wiertz E.J.H.J. (2006). Bovine herpesvirus 1 UL49.5 protein inhibits the transporter associated with antigen processing despite complex formation with glycoprotein M. J. Virol..

[26] Kowal E.J.K., Ter-Ovanesyan D., Regev A., Church G.M., Kuo W.P., Jia S. (2017). Extracellular Vesicle Isolation and Analysis by Western Blotting. Extracellular Vesicles.

[27] McCabe J.B., Berthiaume L.G. (1999). Functional Roles for Fatty Acylated Amino-terminal Domains in Subcellular Localization. MBoC.

[28] Bostanci O., Kartal K., Battal M. (2014). Liver Metastases of Unknown Primary: Malignant Melanoma. Case Rep. Hepatol..

[29] Wu Y., Deng W., Klinke II D.J. (2015). Exosomes: Improved methods to characterize their morphology, RNA content, and surface protein biomarkers. Analyst.

[30] Gangadaran P., Li X.J., Lee H.W., Oh J.M., Kalimuthu S., Rajendran R.L., Son S.H., Baek S.H., Singh T.D., Zhu L. (2017). A new bioluminescent reporter system to study the biodistribution of systematically injected tumor-derived bioluminescent extracellular vesicles in mice. Oncotarget.

[31] de Barros A., Tsourkas A., Saboury B., Cardoso V., Alavi A. (2012). Emerging role of radiolabeled nanoparticles as an effective diagnostic technique. EJNMMI Res..

[32] Gangadaran P., Hong C.M., Ahn B.-C. (2017). Current Perspectives on In Vivo Noninvasive Tracking of Extracellular Vesicles with Molecular Imaging. BioMed Res. Int..

[33] Hostettler L., Grundy L., Käser-Pébernard S., Wicky C., Schafer W.R., Glauser D.A. (2017). The Bright Fluorescent Protein mNeonGreen Facilitates Protein Expression Analysis In Vivo. G3.

[34] Holahan M.R. (2017). A Shift from a Pivotal to Supporting Role for the Growth-Associated Protein (GAP-43) in the Coordination of Axonal Structural and Functional Plasticity. Front. Cell. Neurosci..

[35] Zaborowski M.P., Cheah P.S., Zhang X., Bushko I., Lee K., Sammarco A., Zappulli V., Maas S.L.N., Allen R.M., Rumde P. (2019). Membrane-bound Gaussia luciferase as a tool to track shedding of membrane proteins from the surface of extracellular vesicles. Sci. Rep..

[36] Somasundaram R., Herlyn M. (2012). Melanoma exosomes: Messengers of metastasis. Nat. Med..

[37] Ohno Y., Kihara A., Sano T., Igarashi Y. (2006). Intracellular localization and tissue-specific distribution of human and yeast DHHC cysteine-rich domain-containing proteins. Biochim. Biophys. Acta.

[38] Rocks O., Gerauer M., Vartak N., Koch S., Huang Z.-P., Pechlivanis M., Kuhlmann J., Brunsveld L., Chandra A., Ellinger B. (2010). The Palmitoylation Machinery Is a Spatially Organizing System for Peripheral Membrane Proteins. Cell.

[39] Liang X., Lu Y., Neubert T.A., Resh M.D. (2002). Mass Spectrometric Analysis of GAP-43/Neuromodulin Reveals the Presence of a Variety of Fatty Acylated Species. J. Biol. Chem..

[40] Bijlmakers M., Marsh M. (2003). The on–off story of protein palmitoylation. Trends Cell Biol..

[41] Hou H., John Peter A.T., Meiringer C., Subramanian K., Ungermann C. (2009). Analysis of DHHC Acyltransferases Implies Overlapping Substrate Specificity and a Two-Step Reaction Mechanism. Traffic.

[42] Yang F., Moss L.G., Phillips G.N. (1996). The Illolecular structure of green fluorescent protein. Nat. Biotechnol..

[43] Liu J., Hughes T.E., Sessa W.C. (1997). The first 35 amino acids and fatty acylation sites determine the molecular targeting of endothelial nitric oxide synthase into the Golgi region of cells: A green fluorescent protein study. J. Cell Biol..

[44] Sardar Sinha M., Ansell-Schultz A., Civitelli L., Hildesjö C., Larsson M., Lannfelt L., Ingelsson M., Hallbeck M. (2018). Alzheimer’s disease pathology propagation by exosomes containing toxic amyloid-beta oligomers. Acta Neuropathol..

[45] Venturini A., Passalacqua M., Pelassa S., Pastorino F., Tedesco M., Cortese K., Gagliani M.C., Leo G., Maura G., Guidolin D. (2019). Exosomes From Astrocyte Processes: Signaling to Neurons. Front. Pharmacol..

[46] Tian T., Wang Y., Wang H., Zhu Z., Xiao Z. (2010). Visualizing of the cellular uptake and intracellular trafficking of exosomes by live-cell microscopy. J. Cell. Biochem..

[47] Haertinger M., Weiss T., Mann A., Tabi A., Brandel V., Radtke C. (2020). Adipose Stem Cell-Derived Extracellular Vesicles Induce Proliferation of Schwann Cells via Internalization. Cells.

